# Spanish Translation and Cultural Adaptation of the Wolf Motor Function Test for Survivors of Acquired Brain Injury

**DOI:** 10.3390/healthcare13161969

**Published:** 2025-08-11

**Authors:** Empar Casaña-Escriche, Ángel Sánchez-Cabeza, Elisabet Huertas Hoyas, Desirée Valera-Gran, Eva-María Navarrete-Muñoz

**Affiliations:** 1 Occupational Therapy Research Group (InTeO, Investigación en Terapia Ocupacional), Miguel Hernández University, 03550 San Juan de Alicante, Alicante, Spain; ecasana@umh.es (E.C.-E.); enavarrete@umh.es (E.-M.N.-M.); 2 Rehabilitation, Physiotherapy and Occupational Therapy Service, University Hospital Fundación Alcorcón, 28922 Alcorcón, Madrid, Spain; angel.cabeza@urjc.es; 3 Department of Physiotherapy, Occupational Therapy, Rehabilitation and Physical Medicine, Faculty of Health Sciences, Rey Juan Carlos University, 28933 Móstoles, Madrid, Spain; elisabet.huertas@urjc.es; 4 Institute for Health and Biomedical Research of Alicante (Instituto de Investigación Sanitaria y Biomédica de Alicante, ISABIAL), 03010 Alicante, Alicante, Spain; 5 Department of Surgery and Pathology, Miguel Hernández University, 03550 San Juan de Alicante, Alicante, Spain

**Keywords:** Wolf Motor Function Test, acquired brain injury, upper limb, test adaptation, functionality

## Abstract

**Background/Objectives**: The Wolf Motor Function Test (WMFT) is a standardised assessment tool used to evaluate upper limb (UL) performance in individuals with acquired brain injury (ABI). It consists of 15 timed movement tasks, two strength measures, and a functional ability scale that assesses the quality of movement from 0 to 5. This study aimed to translate and culturally adapt the WMFT for Spanish-speaking individuals with ABI. **Methods**: The translation and cultural adaptation process followed established guidelines and involved researchers from the Rey Juan Carlos University (URJC) and from the Investigación en Terapia Ocupacional (InTeO) group. A joint committee of experts from both research groups unified two previous versions into the final Spanish version of the WMFT. The pilot study included 60 ABI survivors, who were evaluated for the clarity and usability of the adapted test. Descriptive statistical analysis was conducted to evaluate participant characteristics and test performance, with the results summarised for both the less-affected and most-affected UL. **Results**: The final version of the tool features inclusive language and a unified administration procedure. In the pilot study, execution times were longer when using the most-affected UL, particularly for tasks involving object manipulation, while grip strength was lower. **Conclusions**: The Spanish version of the WMFT is a suitable tool for evaluating UL function in ABI survivors and shows promising clinical and research implications.

## 1. Introduction

Acquired brain injury (ABI) is a health condition that significantly impacts an individual’s life. ABI refers to damage that can affect various areas of the brain and is classified into two main types: traumatic brain injury (TBI), resulting from external forces, and non-traumatic brain injury (Non-TBI), caused by internal factors such as stroke or infections [1]. In rehabilitation, approaches to treating TBI and Non-TBI are often similar, with no significant differences in methodologies [2]. In Spain, the prevalence of ABI is reported to be 9.9%, with Non-TBI accounting for 8.9% of the cases, according to a National Institute of Statistics (INE) survey on disability, personal autonomy, and dependency conducted in 2023 [3]. These figures highlight that stroke, a major form of Non-TBI, remains one of the leading causes of long-term disability globally [4].

ABI can result in a wide range of consequences, including cognitive and language impairments, emotional and behavioural changes, and sensory and motor deficits [5]. These challenges can profoundly affect an individual’s daily life and overall well-being. Among these, motor deficits in the lower and upper limbs (ULs) often become a primary focus in rehabilitation [6]. Specifically, the upper limb (UL) is frequently affected, with common limitations including hemiparesis, alterations in muscle tone, and impairments in strength or sensory perception [7]. These deficits often lead to substantial restrictions in daily living activities and limit participation in social and occupational roles [8].

An accurate patient assessment in neurorehabilitation is crucial for individuals with ABI, as it enables the development of tailored intervention strategies and facilitates ongoing progress monitoring [9]. In the field of occupational therapy, the use of standardised assessment tools is essential for ensuring accurate clinical practice. These tools provide precise, objective, and reproducible data, which are fundamental for evaluating outcomes in individuals with ABI and guiding treatment decisions [10]. Consistent evidence from an umbrella review on UL outcome measures for ABI following stroke strongly supports the use of specific assessment tools, including the Fugl–Meyer Assessment, Box and Blocks Test, Chedoke Arm and Hand Activity Inventory, ABILHAND test, and Wolf Motor Function Test (WMFT). These tools are recognised for their high measurement quality and clinical utility [9].

The WMFT is recommended by the European evidence-based recommendations for the clinical assessment of UL in neurorehabilitation (CAULIN) [11]. Moreover, the Spanish consensus among occupational therapists on UL assessment tools for stroke identifies the WMFT as a valid and feasible outcome measure at the activity level, as classified by the International Classification of Functions (ICF). This consensus supports its application for evaluating functional outcomes during recovery treatment [12].

The WMFT is also a cost-effective assessment tool [13] designed to evaluate UL motor capacity through functional tasks and analytical movements [14]. It has been translated and validated in several languages, including French [15], Italian [16], Nepalese [17], and Brazilian Portuguese [18]. Its psychometric properties have been rigorously assessed in studies involving populations with ABI, showing strong intra- and inter-rater reliability, adequate internal consistency, and convergent validity with other motor skill assessment instruments [15,16,17,18].

However, to date, no research has evaluated the validity or psychometric properties of the WMFT in the Spanish-speaking people of ABI. Establishing the feasibility and reliability of the WMFT for this population is a crucial first step. Therefore, the objective of this study was to translate and culturally adapt the WMFT for Spanish-speaking individuals with ABI.

## 2. Materials and Methods

### 2.1. Study Design

The present study is part of InstrumenTO-DCA, an ancillary project within the broader InstrumenTO initiative (https://inteo.umh.es/instrumento/) led by the Research in Occupational Therapy (Investigación en Terapia Ocupacional, InTeO) group at Universidad Miguel Hernández (https://inteo.edu.umh.es/). The primary aim of InstrumenTO-DCA is to translate, culturally adapt, and validate cognitive and manual dexterity assessment tools for Spanish-speaking individuals with ABI. This endeavour aligns with the overarching goals of InstrumenTO project, which is dedicated to the development, translation, adaptation, and validation of occupational therapy assessment instruments. The project aims to ensure that these tools are both linguistically appropriate and culturally relevant for the target population, thereby enhancing their applicability in clinical and rehabilitation settings.

The translation and cultural adaptation of the Spanish version of the WMFT was carried out by researchers from the Rey Juan Carlos University (URJC, its Spanish acronym) and the InTeO group. This pilot study was designed to assess the feasibility of the Spanish version of the WMFT using a cross-sectional approach. Its primary aim was to evaluate the test’s applicability and clarity. Specifically, the study examined potential challenges in administering the test, the comprehensibility of the instructions, the level of prior knowledge required for effective use, and the ease with which patients could understand and follow the instructions.

### 2.2. Participants

The recruitment for this study was conducted in two phases. The first phase took place at Hospital Universitario Fundación Alcorcón (HUFA) (Madrid) between 2018 and 2023, while the second phase occurred in 2024 at the Unidad de Daño Cerebral Hermanas Hospitalarias (Valencia), Hospital General Universitario (Valencia), and the Asociación de Daño Cerebral (ADACEA) (Alicante).

This pilot study targeted adults with ABI who were over 18 years old and clinically stable. Participants were selected by expert therapists who assessed their range of motion, including active joint movement. Specifically, participants needed to demonstrate minimal neuromuscular activation to shoulder flexion and abduction, enabling them to lift their forearm against gravity and place it on a table. Additionally, participants were required to meet the following inclusion criteria: they had to be diagnosed with ABI in the acute, subacute, or chronic stage, demonstrate active participation in a rehabilitation process, and be proficient in the Spanish language. Exclusion criteria included severe comprehension, visual, and auditory deficits that completely impaired their ability to perform the test, as well as UL amputation.

Participants were recruited from neurological rehabilitation centres and assessed in a controlled clinical environment. The sample included individuals with a diagnosis of TBI or Non-TBI, at least one month post-injury. The final sample included 60 participants with ABI, evenly split between InTeO (n = 30) and URJC (n = 30). Sociodemographic data, including gender, age, educational level, and stage of dependence, were collected along with clinical data, such as type and date of diagnosis, hand dominance, and UL impairment. In addition, participants were asked to provide feedback on the difficulty and comprehensibility of the test, contributing valuable insights for further adaptation and validation.

### 2.3. Wolf Motor Function Test (WMFT)

**Figure 1:**
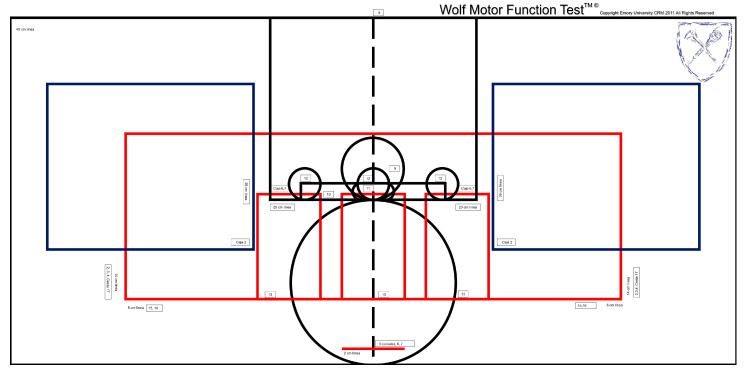
Template showing the placement positions of objects for the WMFT tasks.

The WMFT is a standardised assessment tool commonly used to evaluate manual dexterity in individuals with hemiparesis following an ABI. The tool consists of 17 tasks: 15 measure the time required to complete specific movements, and 2 assess UL strength. To ensure consistency during administration, the template must be centrally positioned on the table, with each task corresponding to a designated space marked by its number, indicating the precise location from where the object is to be picked up. See Figure 1 for a visual representation of the template layout.

**Figure 2:**
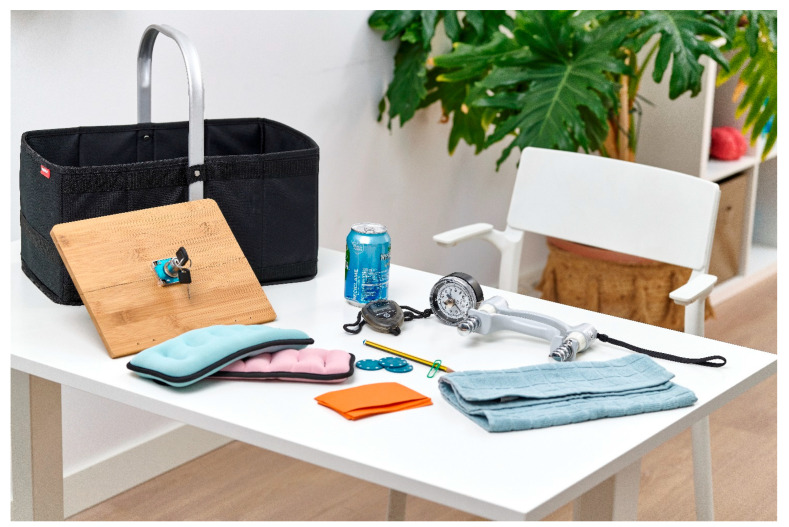
Materials required for the administration of the WMFT.

The first six tasks focus on movements involving joint segments controlling the elbow and shoulder, with performance measured using a stopwatch. Tasks seven and fourteen evaluate strength through shoulder flexion and grip strength, using a dynamometer. The remaining nine tasks assess functional movements, specifically to evaluate grasping abilities [14]. These tasks include picking up objects such as a can, pencil, paperclip, or checkers; flipping cards; turning a key in a lock; folding a towel; and lifting a basket. See Figure 2 for the complete set of materials used in the WMFT.

Scoring is based on multiple performance characteristics, including speed, functionality, accuracy, fluency, and coordination with everyday objects. The assessment is scored using two parameters: timed duration and functional ability (FA) [13]. The execution time for each item is limited to a maximum of 120 s. FA is scored on a scale of 0 to 5, where 0 indicates “no attempt with the UL” and 5 represents “normal movement”, reaching a maximum score of 75 points. Therefore, lower FA scores and longer execution times reflect poorer UL functionality [14].

### 2.4. Administration Procedure

The WMFT was administered following standardised procedures to ensure consistency and accuracy. The test setup included the following:

- Test set-up: Adequate space was arranged in the test room to ensure the participant’s comfort, as well as proper chair and recording positions. It was essential that we ensure that the tested movement was clearly visible. As specified in the original version of the test, the administration was performed first with the less-affected UL and then with the most-affected UL.
- Instructions: General instructions were provided prior to the test administration. Each item was described and demonstrated twice: the first demonstration was performed slowly to familiarise the participant with the task, while the second demonstration was faster to show the required speed. Participants were not allowed to practice the tasks before the test commenced.
- Execution: The test was administered in a quiet environment to minimise distractions. Participants were encouraged to complete each task as quickly as possible and to achieve the highest weight as possible in the strength tasks. A second trial of the task was only allowed if the participant performed the task incorrectly, if there was a distraction or interruption, if the assessor made an error in preparation or timing, or if an object was dropped on the floor for more than 5 s. A second trial was not permitted if the assessor believed the participant could perform the task better or faster.
- Scoring: The time taken to complete each task was recorded using a stopwatch. The FA score was determined by an expert panel after viewing the recorded sessions, as specified in the instructions. The panel followed the Functional Ability Scale (FAS) and the scoring guidelines for each task.

### 2.5. Process of Translation and Cultural Adaptation of the WMFT into Spanish

Translation refers to the linguistic process of converting the content of an assessment tool from one language to another, ensuring semantic equivalence. In contrast, cultural adaptation involves modifying the instrument to ensure that its content, format, and administration procedures are appropriate and meaningful within the cultural context of the target population. This may include changes to terminology, examples, measurement units, and visual materials. This step is essential for preserving the conceptual and functional equivalence of the original tool. It ensures that the instrument is not only linguistically accurate but also culturally relevant and valid for the population it is intended to assess. In our study, this process was guided by internationally recognised frameworks [19,20,21], and it was crucial that we ensure that the Spanish version of the WMFT would be both understandable and applicable to Spanish-speaking individuals with ABI.

**Figure 3:**
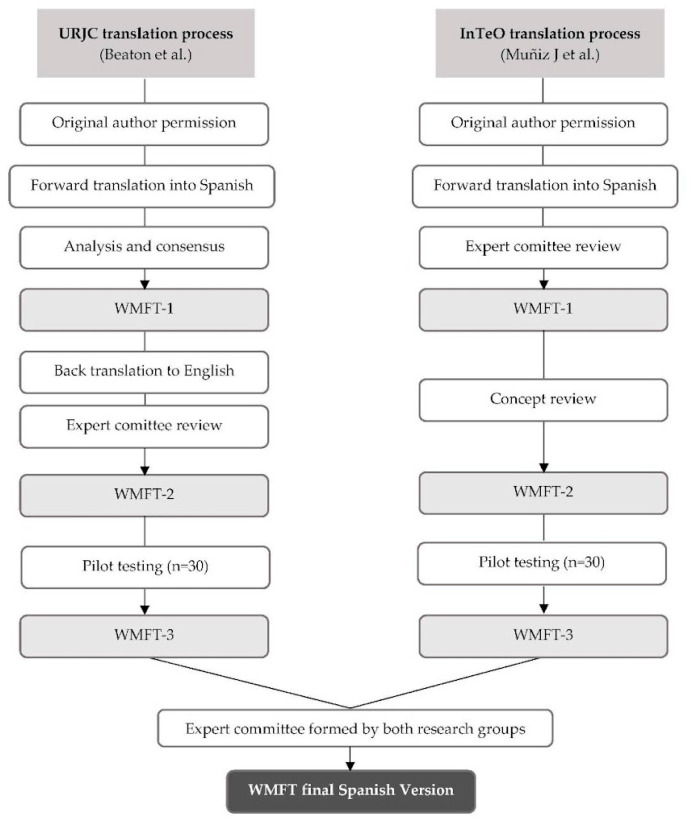
Flowchart of the translation and cross-cultural adaptation process.

The translation and cultural adaptation of the original version of the WMFT into Spanish were carried out in three phases. Figure 3 illustrates this process through a flowchart, providing a visual summary of the steps followed. The URJC and the InTeO group obtained permission from the author for the translation and validation of the test for the Spanish population. Each research group independently conducted its own translation and adaptation process, after which both versions were combined. A standardised procedure was followed in both processes, although back-translation was not used. Previous studies have shown that a linguistically proficient expert committee can ensure a direct and high-quality translation without the need for back-translation [22,23].

The translation and cultural adaptation of the WMFT involved several expert panels across different phases of the process. These panels were composed of professionals with diverse backgrounds to ensure linguistic accuracy, clinical relevance, and conceptual equivalence. The URJC expert committee included four occupational therapists with expertise in neurology (each with at least two years of clinical and research experience and formal training in neurorehabilitation), two professional translators, and two researchers with prior experience using the WMFT. The InTeO expert committee comprised four occupational therapists specialised in neurology (each with at least two years of clinical and research experience) and two methodologists with academic backgrounds in occupational therapy. Additionally, a conceptual review was conducted by three neurorehabilitation experts with no prior experience using the WMFT, to ensure clarity and neutrality. Finally, a joint expert committee composed of the authors of this article was responsible for reconciling the two versions. This panel included three occupational therapists (two with over twenty years of clinical and research experience in neurology, and one with two years), and two methodologists with over ten years of experience in occupational therapy research.

The URJC translation process was conducted according to the methodology outlined by Beaton et al. [19] and followed these steps:

1. Translation into Spanish: The test was translated by two native Spanish-speaking translators, resulting in translations T1 and T2.
2. Analysis and consensus: Both translations were reviewed and analysed to reach a consensus on a single version (T1-2).
3. Back translation to English: Native English-speaking translators, unaware of the original translation, independently translated T1-2 back into English (RT1 and RT2).
4. Expert committee review: The committee, composed of four occupational therapists, two translators, and two researchers familiar with the tool, reviewed all the versions (T1, T2, T1-2, RT1, and RT2). After a thorough analysis, a prefinal Spanish version was selected.
5. Pilot testing: The prefinal version was tested with a sample of 30 participants, who were interviewed about any difficulties in understanding the meaning of the questions and the responses. Incidents of incomplete or repeated responses were also noted (i.e., when all patients provide the same response to a specific question).
6. Final revision: Errors and typographical mistakes were corrected, leading to the final version of the test, which was sent to the authors of the original version.

The InTeO translation process followed the methodology published by Muñiz et al. [20] and included the following steps:

1. Translation into Spanish: Two native Spanish-speaking translators independently translated the test, resulting in translations T1 and T2.
2. First expert committee review: A committee of four occupational therapists with expertise in neurology and two methodologists met six times to review and unify the two translations. They made semantic and idiomatic adjustments to create the first Spanish version (WMFT-E-1).
3. Concept review: Three neurorehabilitation experts conducted a content analysis and administered the test to identify any comprehension issues across the test’s instructions, resulting in the second version of the tool (WMFT-E-2).
4. Pilot study: WMFT-E-2 was tested with a sample of participants with ABI. Participants were interviewed about any difficulties they encountered with the translated WMFT version. Evaluators also noted any questions that caused difficulty during the assessment, and the FA expert panel proposed changes to refine the administration and scoring instructions.
5. Final version: Changes based on the pilot study feedback were incorporated, resulting in the final version of the test, which was translated and culturally adapted into Spanish (WMFT-E-3).

Finally, the expert committee compared the two pre-final versions, discussed discrepancies, and reached a unified version. The final Spanish version of the WMFT incorporated inclusive language, reorganised instructions, standardised terminology, and included visual aids to enhance clarity. Although no separate table was created for the unified version, the issues addressed during each group’s process are detailed in Table 1 (URJC) and Table 2 (InTeO). The final version was informed by both expert consensus and pilot testing with individuals with ABI.

### 2.6. Pilot Testing of the Spanish Version of the WMFT

A pilot study was conducted with 60 ABI survivors to evaluate the translated and adapted version of the WMFT. The tool was administered by three evaluators: one from URJC in Madrid and two from InTeO group in Valencia and Alicante. During the test administration, assessors recorded any issues related to comprehension and scoring issues. At the end of the test, participants were asked to report any difficulties they encountered during the session. The primary aim was to identify areas for improvement before finalising the version.

### 2.7. Ethical Considerations

This research was approved by the ethics committee of the General University Hospital of Alicante (Acta 2023-08) and the Rey Juan Carlos University Ethics Committee (1602201703517). All procedures were conducted in accordance with the Declaration of Helsinki and written informed consent was provided by all participants.

### 2.8. Statistical Analysis

Statistical analysis was performed using R software version 4.2.2 (R Foundation for Statistical Computing, Viena, Austria; http://www.r-project.org). As this was a feasibility and pilot study, the analysis was limited to descriptive statistics to explore the preliminary performance of the Spanish version of the WMFT in individuals with ABI. Demographic and clinical characteristics of the participants were summarised using frequencies and percentages (n, %) for the categorical variables. To assess the normality of the quantitative variables, the Shapiro–Wilk test was applied. For normally distributed quantitative variables, mean and standard deviation (mean, SD) were reported, while, for variables not following a normal distribution, median and interquartile range (median, IQR) were used.

Time in seconds for all items was summarised by left and right UL, distinguishing between the less-affected and most-affected limb. FA scores, on a scale from 0 to 5, were only assessed for the most-affected limb, with results presented for both the left and right sides.

## 3. Results

### 3.1. Consensus-Based Revisions in the Cross-Cultural Adaptation of the WMFT

Table 1 and Table 2 highlight the most significant discrepancies identified during the translation and cultural adaptation process by the URJC team and the InTeO group, respectively. Following a joint review by both research groups, a consensus was reached to introduce modifications across the full text to make the language more inclusive and aligned with contemporary usage. Key changes included replacing “paciente” (patient) with “persona evaluada” (participant) and “evaluador” (evaluator) with “persona evaluadora” (assessor). Additionally, the terminology for describing UL was revised: “extremidad superior no afectada” (unaffected upper extremity) was changed to “extremidad superior menos afectada” (less-affected UL) and “extremidad superior afectada” (affected upper extremity) was changed to “extremidad superior más afectada” (most-affected UL). These revisions align with the terminology commonly used in the current literature.

To improve the comprehension and usability of the WMFT, the instructions were reorganised into a logical sequence, and the terminology was standardised. Furthermore, images were incorporated to visually support the instructions, and the double demonstration was preserved, enhancing clarity for users of the tool. The committee also emphasised that assessors should possess knowledge about UL biomechanics and anatomy, ensuring they are well-prepared to apply the tool effectively. After implementing these modifications, participants reported no difficulties in understanding the WMFT instructions. Feedback from the expert committee confirmed that the revised language was appropriate for the target audience, further validating the effectiveness of the adaptation process. All modifications to the WMFT instructions were reviewed and approved by the original author of the test. The author agreed with the proposed changes, which aimed to improve clarity and usability for Spanish-speaking assessors and participants. No additional comments or objections were raised during this consultation.

### 3.2. Sample Description

Table 3 displays the sociodemographic characteristics of the participants from the URJC and InTeO samples. Among the 60 participants, 38 (63.3%) were men, with a notably higher proportion of men in the URJC sample compared to InTeO group (56.7%). The average age of participants was 61 years (SD = 14.2), with the URJC sample being older on average (64 years, SD = 11.9) compared to the InTeO sample (58 years, SD = 15.9). Regarding education level, 56.7% of participants from both the URJC and InTeO samples had more than a primary education, with no significant difference in educational attainment between the samples. Although a large proportion of participants reported no caregiver support overall (83.3%), we observed differences between the samples: 93.3% of the URJC sample reported no caregiver support, compared to 73.3% in the InTeO group. All participants were right-handed, and UL impairments were almost equally distributed laterally. However, the proportion of participants with right UL impairments were higher in the URJC (63.3%), while the InTeO group had a slightly greater proportion with left UL impairments (53.3%). The most common type of ABI was Non-TBI, accounting for 91.7% of the total sample. The URJC sample consisted entirely of Non-TBI cases (100%), while the InTeO sample included 10% of participants with TBI. The main difference observed between the two samples was in the time from ABI onset to the test session. The URJC sample had a median of 1.6 months (IQR: 1.0–1.4), while the InTeO sample had a median of 5.6 months (IQR: 3.3–14.7).

### 3.3. Pilot Testing Results

Table 4 and Table 5 display the results of the pilot testing of the WMFT for the URJC and InTeO samples. Table 4 presents the median and IQR for the execution times and strength scores (in kg) for the 17 WMFT tasks performed with the less-affected UL. Overall, the execution times with the less-affected right UL was slightly higher for the URJC sample (41.7 s; IQR: 35.9–44.8) compared to the InTeO sample (40.0 s; IQR: 33.0–69.8). In contrast, execution times for the less-affected left UL was slightly higher for the InTeO sample (41.1 s, IQR: 33.3–47.0) compared to the URJC sample (39.9 s; IQR: 35.2–49.5). Regarding strength, notable differences in grip strength were observed between the two samples. The URJC sample showed a higher median grip strength for the left UL (38.3 kg; IQR: 27.9–40.1), while the InTeO group had a median of 29.3 kg (IQR: 23.3–37.3). Conversely, grip strength for the right UL was higher in the InTeO sample (31.6 kg; IQR: 20.3–38.0) compared to the URJC sample (28.0 kg; IQR: 25.3–35.4).

Table 5 presents the results for the most-affected UL, including execution times, weight scores, and FA scores for the 17 WMFT tasks. The results revealed that total median FA scores were higher in the InTeO sample than the URJC sample in both ULs. Overall, the execution time was shorter when performed with the right UL in both samples.

## 4. Discussion

This study presents a translated and culturally adapted version of the WMFT for the Spanish population. The adaptation process followed a standardised methodology, with slight variations between the two research teams. The URJC researchers followed a detailed process to develop the pre-final version of the WMFT, which included a direct translation, a review by an expert committee, back-translation, a pilot study, and final revisions. In contrast, the InTeO group implemented a similar approach but omitted the back-translation step, in line with the guidelines proposed by Muñiz et al. (2013) [20]. Instead, they incorporated an additional conceptual review by three external neurorehabilitation specialists. The use of two distinct translation procedures reflects the independent timelines and institutional contexts of the URJC and InTeO research teams. Each group received separate authorisation from the original author and followed established guidelines appropriate to their respective methodologies. This dual approach enriched the adaptation process and allowed for a broader expert consensus in the final version. Despite these differences, the final version of the WMFT was merged through expert collaboration, ensuring linguistic accuracy and cultural relevance for the Spanish-speaking population with ABI.

Although we based our adaptation process on well-established guidelines by Muñiz et al. (2013) [20] and Beaton et al. (2000) [19], the Spanish version of the WMFT also meets the test adaptation requirements outlined by Hernández et al. (2020) [21]. During the adaptation process, we ensured that the test instructions and item content conveyed similar meaning for the target population (TD3) [21], with particular attention to clarity and cultural relevance and an emphasis on using more inclusive language [24]. To achieve this, the “General comments” section was reorganised, and the terminology was unified to enhance comprehension and equity (TD3-1 and TD3-2) [21]. The item formats, response options, scoring rubrics, and administration procedures were carefully adapted to align with the original version (TD4) [21]. We ensured that the procedures were familiar and suitable for the target population (TD4-1 and TD4-2) [21], making the test accessible and practical for evaluators. A key addition to the adapted version was the recommendation for evaluators to have prior knowledge of biomechanics and anatomy, in line with the original author’s guidance on test application. Additionally, photographs were included to clarify the tasks and further support evaluators.

To assess the suitability of the Spanish version of WMFT, a pre-testing phase was conducted with a sample of participants with ABI. While the recommended sample size for pilot testing typically ranges from 10 to 40 participants [25], we conducted the pre-test with 60 ABI participants to ensure a more robust assessment. Following the best practices for improving the validity of health measurements [24,26], cognitive interviews were carried out with each participant to rate the clarity of the WMFT instructions and items using a dichotomous scale (“clear” or “unclear”). The evaluators concluded that the instructions and task descriptions were generally clear, and the process proceeded smoothly. However, several participants experienced difficulties in task 15 “Turning the key in the lock”. This task was not modified, as the original author had already accounted for potential cognitive challenges faced by individuals with ABI. During the pre-test, evaluators provided verbal assistance when necessary and assigned a lower FA score if the task was poorly performed. For the remaining tasks, no comprehension issues were reported, as they were simpler, and the double demonstration was sufficient to ensure understanding. Moreover, the pilot study revealed that educational level did not significantly affect participants’ understanding of the WMFT instructions, supporting the instrument’s accessibility across diverse educational backgrounds.

In addition to evaluating the clarity of the WMFT instructions, another important aspect of the pilot test was assessing the suitability of the tool for measuring UL function in individuals with ABI. This pilot testing provided valuable insights into the manual dexterity of participants by evaluating both their most and less-affected UL. The results showed that the WMFT can be sensitive to differences in UL function across both limbs, with the performance varying depending on laterality and stage of recovery. The differences observed in the performance of the two groups, URJC (subacute condition) and InTeO (chronic condition), further supported the reliability of the instrument in capturing the impact of ABI on UL function. Specifically, individuals in the chronic stage, who had undergone more rehabilitation, tended to perform better, showing greater dexterity and higher FA scores compared to those in the subacute stage. These findings suggest that the WMFT can be sensitive to varying stages of recovery [27], and can reliably measure changes in UL function over time, reinforcing its value in assessing manual dexterity in individuals with ABI. Moreover, the pilot study revealed that laterality (hand dominance) played a role in task performance. While right-handed participants generally performed better with their dominant right UL, overall execution times were shorter for the right UL across both samples, supporting the influence of hand dominance in task performance. However, the InTeO sample showed better recovery in the left UL, further highlighting the potential impact of rehabilitation in restoring function in the less-dominant hand. This finding emphasises the importance of considering both the most- and less-affected limbs when assessing UL function in individuals with ABI.

The availability of an inclusive and culturally adapted Spanish version of the WMFT represents a significant step toward harmonising outcome measurement in neurorehabilitation across Spanish-speaking regions. Its standardised administration and scoring procedures, combined with inclusive terminology, enhance its applicability in diverse clinical and research settings. This facilitates its use in multi-centre clinical trials, cross-site benchmarking, and collaborative studies aimed at evaluating the effectiveness of interventions for UL recovery after ABI. The Spanish version also contributes to the development of shared outcome frameworks and may support the generation of comparable data across institutions and countries. Future validation studies will further explore the tool’s psychometric properties, including its sensitivity to change and applicability across different stages of recovery. Additionally, future exploration could consider integrating the Spanish WMFT into digital rehabilitation platforms or combining it with AI-based movement analysis to further expand its clinical applications and research impact, as suggested by the recent advancements in AI-enhanced motor assessment [28].

This study has several limitations that should be considered. One key limitation is the exclusion of participants in the acute stage of ABI, which could offer additional insights into the tool’s applicability across all recovery stages. Additionally, future studies should aim to include a broader range of socio-demographic samples to improve generalizability.

A key strength of this study lies in the rigorous adaptation process following well-established procedures for instrument translation and cultural adaptation, The process involved standardised methodologies that ensured linguistic, cultural, and procedural adjustments were made to guarantee the tool’s accessibility and relevance for Spanish-speaking individuals with ABI. These modifications were designed to facilitate the administration and understanding of the WMFT for both evaluators and participants. Finally, the pilot confirmed the effectiveness and cultural relevance of the Spanish version of the WMFT, showing that it is a reliable tool for assessing UL function in individuals with ABI.

## 5. Conclusions

This study provides a translated and culturally adapted Spanish version of the WMFT for professionals working with Spanish-speaking individuals with ABI. The pilot test confirmed that the tool is comprehensible and usable for both evaluators and participants, ensuring its potential for effective implementation in clinical practice. The WMFT shows great promise for improving the evaluation of functional tasks and motor skills, ultimately enhancing rehabilitation strategies and patient outcomes. Future research should focus on evaluating the psychometric properties of the tool to further establish its effectiveness in clinical practice with Spanish ABI survivors. 

## Figures and Tables

**Table 1:** Analysis and resolutions by the URJC expert committee during the translation and cultural adaptation of the WMFT instructions.

Instructions	Problem	Solution
Objects	Objects used were culturally appropriate for the Spanish context, but the units of force were different.	The units of force were changed from pound (lbs.) to kilograms (kg).
General information	The original text lacked a structure that facilitated comprehension.	The instructions were reorganised into a logical sequence to enhance understanding. This restructuring clarified the purpose of the test, the administration process, the scoring method, and the recording procedures.
Individual tasks instructions	Instructions were complex.	Images were incorporated to enhance the clarity and understanding of the instructions.

**Table 2:** Analysis and resolutions by the InTeO expert committee during the translation and cultural adaptation of the WMFT instructions.

Instructions	Problem	Solution
Language	The language used was not inclusive. The terms “paciente” (patient) and “evaluador” (evaluator) were used, and the terms “extremidad superior no afectada” (unaffected upper extremity) and “extremidad superior afectada” (affected upper extremity) were used to describe the limbs.	“Patient” and “evaluator” were replaced with “persona evaluada” (participant) and “persona evaluadora” (assessor) to ensure inclusivity. “Extremidad superior no afectada” (unaffected upper extremity) and “extremidad superior afectada” (affected upper extremity) were replaced with “extremidad superior menos afectada” (less-affected UL) and “extremidad superior más afectada” (most-affected UL) for greater clarity and sensitivity.
Objects	List of objects was presented without accompanying images.	An image was included to illustrate the required objects.
General information	The text was excessively lengthy, complex, and repetitive, making it difficult to read.	Repetitive instructions were unified and reorganised to enhance clarity and facilitate comprehension.
	Some evaluators were not prepared to understand the instructions.	The requirement was added: “To be an evaluator, you should have knowledge about UL biomechanics and anatomy”.
	The double instruction (slow and fast) and the demonstration were eliminated.	It was clarified that the instruction and demonstration could be repeated as needed.
Individual tasks instructions	The instructions presented significant challenges in terms of comprehension.	Terminology was unified, and items were organised into a consistent structure, making the administration process faster.
	The instruction was reiterated twice, first at a slow pace and then at a faster pace.	The repeated instruction was removed to streamline the process.
Filming position	The movement was not clearly visible.	The camera position was modified to ensure better visibility of the movement.
Print instructions	The information was lengthy and irrelevant to the context in Spain.	All information, except the template dimensions, was eliminated.

Abbreviations: UL, upper limb.

**Table 3:** Sociodemographic and clinical characteristics of the participants in the pilot testing (n = 60).

	URJC Sample	InTeO Sample	Total Sample
	(n = 30)	(n = 30)	(n = 60)
Sex, n (%)			
Women	9 (30)	13 (43.3)	22 (36.7)
Men	21 (70)	17 (56.7)	38 (63.3)
Age, mean (SD)	64 (11.9)	58 (15.9)	61 (14.2)
Education level, n (%)			
≤Primary education	13 (43.3)	13 (43.3)	26 (43.3)
>Primary education	17 (56.7)	17 (56.7)	34 (56.7)
Patient with a caregiver, n (%)			
Yes	2 (6.7)	8 (26.7)	10 (16.7)
No	28 (93.3)	22 (73.3)	50 (83.3)
Most-affected UL, n (%)			
Left	11 (36.7)	16 (53.3)	27 (45.0)
Right	19 (63.3)	14 (46.7)	33 (55.0)
Type of ABI, n (%)			
Non-TBI	30 (100)	25 (83.4)	55 (91.7)
TBI	-	3 (10.0)	3 (5.0)
Other	-	2 (6.6)	2 (3.3)
Months from ABI onset to the test session, median (IQR)	1.6 (1.0, 1.4)	5.6 (3.3, 14.7)	2 (1.2, 5.5)

Abbreviations: InTeO, Investigación en Terapia Ocupacional; URJC, Universidad Rey Juan Carlos; ABI, acquired brain injury; UL, upper limb; n, number of participants; % percentage; SD, standard deviation; IQR, interquartile range.

**Table 4:** Median and interquartile range of time in seconds and strength in kg for WMFT tasks on the less-affected UL in the pilot study (n = 60).

	URJC Sample		InTeO Sample		Total Sample	
Tasks (Time)	Left UL (n = 19)	Right UL (n = 11)	Left UL (n = 14)	Right UL (n = 16)	Left UL (n = 33)	Right UL (n = 27)
1. Forearm to table (side)	0.7 (0.6; 0.8)	0.7 (0.6; 0.7)	0.9 (0.8; 1.2)	1.0 (0.7; 1.3)	0.8 (0.7; 0.9)	0.7 (0.7; 1.1)
2. Forearm to box (side)	0.9 (0.9; 1.0)	0.9 (0.8; 0.9)	1.3 (1.1; 1.5)	1.1 (0.9; 1.4)	1.0 (0.9; 1.3)	0.9 (0.8; 1.2)
3. Extend elbow (side)	0.9 (0.8; 1.0)	0.9 (0.8; 1.2)	1.3 (0.9; 1.6)	1.0 (0.8; 1.3)	1.0 (0.9; 1.3)	0.9 (0.8; 1.2)
4. Extend elbow (weight)	1.1 (1.0; 1.2)	1.0 (1.0; 1.5)	1.0 (0.9; 1.6)	0.9 (0.7; 1.4)	1.1 (1.0; 1.3)	1.0 (0.9; 1.5)
5. Hand to table (front)	0.7 (0.6; 0.8)	0.7 (0.6; 0.8)	1.0 (0.8; 1.2)	0.9 (0.8; 1.1)	0.8 (0.7; 1.0)	0.8 (0.7; 1.0)
6. Hand to box (front)	0.9 (0.8; 1.0)	1.0 (0.8; 1.3)	1.0 (0.7; 1.2)	0.8 (0.7; 1.0)	0.9 (0.7; 1.0)	0.9 (0.7; 1.1)
7. Weight to box, in kg	4.5 (3.5; 5.5)	5.0 (4.3; 5.0)	5.0 (4.1; 6.1)	5.0 (3.9; 7.0)	5.0 (3.5; 5.5)	5.0 (4.0; 5.3)
8. Reach and retrieve	1.1 (1.0; 1.4)	1.3 (1.0; 1.5)	1.1 (0.9; 1.4)	1.1 (0.9; 1.6)	1.1 (1.0; 1.4)	1.1 (0.9; 1.6)
9. Lift can	1.7 (1.4; 1.9)	1.7 (1.2; 1.8)	1.5 (1.4; 2.0)	1.7 (1.3; 2.0)	1.6 (1.4; 1.9)	1.6 (1.3; 1.9)
10. Lift pencil	1.6 (1.1; 2.0)	1.6 (1.2; 1.8)	1.6 (1.3; 1.9)	1.5 (1.1; 1.8)	1.6 (1.3; 1.9)	1.6 (1.2; 1.8)
11. Lift paper clip	1.9 (1.8; 2.0)	1.9 (1.6; 2.0)	1.6 (1.4; 1.8)	1.5 (1.3; 2.4)	1.8 (1.5; 2.0)	1.7 (1.4; 2.3)
12. Stack checkers	3.9 (3.4; 4.3)	4.0 (3.7; 4.3)	3.7 (3.1; 4.5)	3.9 (3.3; 4.2)	3.8 (3.2; 4.3)	4.0 (3.4; 4.3)
13. Flip cards	6.0 (5.4; 6.7)	6.0 (5.2; 7.0)	7.4 (5.7; 9.8)	5.6 (4.7; 9.3)	6.1 (5.6; 8.6)	5.8 (4.9; 8.8)
14. Grip strength, in kg	38.3 (27.9; 40.1)	28.0 (25.3; 35.4)	29.3 (23.3; 37.3)	31.6 (20.3; 38.0)	35.6 (24.0; 39.5)	29.3 (21.9; 37.1)
15. Turn key in lock	6.4 (5.9; 8.6)	7.3 (6.3; 9.9)	5.1 (4.0; 5.7)	4.9 (4.2; 11.6)	5.9 (5.1; 7.2)	6.4 (4.7; 11.2)
16. Fold towel	8.3 (7.1; 13.4)	8.0 (6.4; 9.9)	6.7 (5.3; 7.6)	7.6 (4.6; 9.7)	7.4 (6.4; 9.2)	7.8 (5.8; 9.2)
17. Lift basket	3.6 (3.0; 3.9)	3.2 (3.0; 3.6)	2.6 (2.1; 3.1)	2.8 (2.2; 3.3)	3.1 (2.6; 3.7)	3.0 (2.6; 3.6)
Total	39.9 (35.2; 49.5)	41.7 (35.9; 44.8)	41.1 (33.3; 47.0)	40.0 (33.0; 69.8)	39.9 (34.2; 48.8)	40.6 (33.3; 53.8)

Abbreviations: WMFT. Wolf Motor Function Test; URJC. Universidad Rey Juan Carlos; InTeO. Investigación en Terapia Ocupacional; UL. upper limb.

**Table 5:** Median and interquartile range of time in seconds, strength in kg, and FA on a scale of 0 to 5 for WMFT tasks on the most-affected UL in the pilot study (n = 60).

Tasks	URJC Sample		InTeO Sample		Total Sample	
	Left UL (n = 11)	Right UL (n = 19)	Left UL (n = 16)	Right UL (n = 14)	Left UL (n = 27)	Right UL (n = 33)
1. Forearm to table (side)						
Time	1.8 (1.3; 2.5)	2.0 (1.6; 2.8)	1.3 (0.9; 1.6)	1.3 (1.0; 1.6)	1.4 (1.0; 2.1)	1.6 (1.3;2.2)
FA	4.0 (4.0; 4.0)	4.0 (3.5; 4.0)	5.0 (4.0; 5.0)	5.0 (4.0; 5.0)	4.0 (4.0; 5.0)	4.5 (4.0; 5.0)
2. Forearm to box (side)						
Time	1.9 (1.9; 2.6)	2.0 (1.9; 2.2)	1.5 (1.1; 2.0)	2.0 (1.6; 2.3)	1.8 (1.3; 2.4)	2.0 (1.7; 2.2)
FA	4.0 (3.5; 4.0)	4.0 (3.5; 4.0)	4.0 (4.0; 5.0)	4.0 (4.0; 4.0)	4.0 (4.0; 4.0)	4.0 (4.0; 4.0)
3. Extend elbow (side)						
Time	2.3 (2.0; 3.6)	2.4 (1.9; 3.5)	1.4 (1.0; 1.8)	1.6 (1.3; 1.9)	1.9 (1.3; 2.6)	2.0 (1.7; 2.6)
FA	3.0 (3.0; 3.5)	4.0 (3.0; 4.0)	4.0 (3.0; 5.0)	4.0 (4.0; 5.0)	3.0 (3.0; 4.0)	4.0 (3.0; 4.0)
4. Extend elbow (weight)						
Time	3.3 (3.0; 4.3)	3.0 (2.7; 3.8)	1.2 (0.9; 1.2)	1.3 (1.1; 2.0)	2.2 (1.1; 3.8)	2.7 (1.5; 3.2)
FA	3.0 (3.0; 3.5)	4.0 (3.0; 4.0)	4.0 (3.8; 5.0)	4.0 (4.0; 5.0)	4.0 (3.0; 4.0)	4.0 (3.0; 4.0)
5. Hand to table (front)						
Time	1.2 (1.0; 1.4)	1.2 (1.0; 1.8)	1.1 (0.9; 1.4)	1.3 (1.1;1.6)	1.1 (0.9; 1.4)	1.2 (1.0; 1.7)
FA	4.0 (4.0; 4.0)	4.0 (4.0; 4.0)	4.5 (3.8; 5.0)	4.5 (4.0; 5.0)	4.0 (4.0; 5.0)	4.0 (4.0; 5.0)
6. Hand to box (front)						
Time	2.0 (1.9; 2.0)	1.9 (1.3; 2.2)	1.0 (0.8; 1.4)	1.1 (0.9; 1.6)	1.4 (0.9; 1.9)	1.6 (1.1; 2.0)
FA	3.0 (3.0; 4.0)	4.0 (3.0; 4.0)	4.0 (4.0; 5.0)	4.0 (4.0; 5.0)	4.0 (3.0; 4.5)	4.0 (3.0; 4.0)
7. Weight to box, in kg	3.0 (2.3; 3.5)	3.5 (2.8; 3.8)	3.5 (2.4; 7.0)	3.5 (2.0; 4.0)	3.0 (2.3; 4.8)	3.5 (2.0; 4.0)
8. Reach and retrieve						
Time	1.9 (1.6; 2.0)	2.0 (1.9; 2.1)	1.4 (1.2; 1.7)	1.4 (1.2; 2.3)	1.6 (1.3; 2.0)	1.9 (1.4; 2.1)
FA	4.0 (3.5; 4.0)	4.0 (3.5; 4.0)	4.0 (3.0; 5.0)	4.0 (4.0; 5.0)	4.0 (3.0; 4.5)	4.0 (4.0; 4.0)
9. Lift can						
Time	2.9 (2.5; 3.1)	2.9 (2.5; 3.4)	2.5 (1.7; 3.0)	2.7 (2.1; 3.6)	2.6 (2.0; 3.1)	2.8 (2.4; 3.5)
FA	3.0 (3.0; 4.0)	4.0 (3.0; 4.0)	4.0 (4.0; 5.0)	4.0 (4.0; 5.0)	4.0 (3.0; 4.5)	4.0 (3.0; 4.0)
10. Lift pencil						
Time	5.0 (2.9; 6.6)	5.0 (4.1; 5.3)	2.0 (1.6; 2.4)	2.4 (1.8; 3.2)	2.7 (2.0; 4.8)	4.1 (2.4; 5.2)
FA	3.0 (3.0; 4.0)	3.0 (3.0; 3.0)	4.5 (4.0; 5.0)	4.0 (4.0; 5.0)	4.0 (3.0; 5.0)	3.0 (3.0; 4.0)
11. Lift paper clip						
Time	8.5 (7.5; 10.5)	8.7 (7.0; 10.7)	2.5 (1.6; 3.6)	3.3 (2.1; 4.0)	4.6 (2.5; 8.9)	6.2 (3.6; 9.5)
FA	3.0 (2.0; 3.0)	2.0 (1.0; 3.0)	4.5 (3.8; 5.0)	4.0 (4.0; 5.0)	4.0 (3.0; 5.0)	3.0 (2.0; 4.0)
12. Stack checkers						
Time	10.8 (9.5; 13.5)	9.6 (8.4; 12.2)	7.4 (3.6; 12.8)	6.2 (5.2; 7.4)	10.6 (6.2; 13.5)	8.5 (5.9; 11.0)
FA	3.0 (3.0; 3.0)	3.0 (3.0; 3.0)	4.0 (2.0; 5.0)	4.0 (4.0; 4.8)	3.0 (3.0; 4.5)	3.0 (3.0; 4.0)
13. Flip cards						
Time	18.6 (14.1; 20.7)	17.5 (11.5; 20.5)	7.3 (5.4; 19.1)	6.2 (5.2; 7.4)	13.8 (6.7; 20.0)	11.5 (9.8; 18.9)
FA	3.0 (3.0; 3.0)	3.0 (3.0; 3.0)	3.0 (3.0; 4.0)	3.0 (3.0; 3.8)	3.0 (3.0; 3.5)	3.0 (3.0; 3.0)
14. Grip strength, in kg	10.0 (8.0; 12.5)	12.5 (8.8; 13.3)	19.0 (10.2; 26.6)	20.2 (12.5; 30.6)	12.5 (9.1; 23.0)	12.5 (9.0; 18.3)
15. Turn key in lock						
Time	20.5 (19.1; 23.0)	17.4 (16.1; 22.9)	8.8 (4.7; 21.3)	6.0 (5.4; 7.6)	18.3 (7.3; 23.0)	14.3 (6.5; 18.7)
FA	3.0 (3.0; 3.0)	3.0 (3.0; 3.0)	3.5 (3.0; 4.0)	3.0 (3.0; 4.0)	3.0 (3.0; 4.0)	3.0 (3.0; 4.0)
16. Fold towel						
Time	12.3 (10.8; 17.4)	15.4 (9.6; 17.5)	8.3 (4.9; 9.8)	7.7 (5.7; 29.1)	9.9 (7.7; 12.4)	10.4 (7.9; 16.0)
FA	4.0 (3.0; 4.0)	4.0 (3.0; 4.0)	4.0 (4.0; 5.0)	4.0 (4.5; 5.0)	4.0 (3.0; 4.0)	4.0 (3.0; 4.0)
17. Lift basket						
Time	9.6 (7.7; 22.9)	8.2 (6.5; 16.8)	3.2 (2.2; 4.9)	3.5 (3.0; 4.3)	5.3 (2.9; 17.5)	5.3 (3.5; 8.3)
FA	4.0 (3.5; 4.0)	4.0 (3.0; 4.0)	4.0 (3.8; 5.0)	4.0 (3.3; 4.0)	4.0 (3.5; 4.0)	4.0 (3.0; 4.0)
Total						
Time	113.0 (93.0; 125.8)	98.8 (84.7; 109.5)	120.1 (34.9; 212.9)	51.3 (45.1; 60.3)	112.95 (65.9; 181.4)	79.4 (57.1; 101.7)
FA	51.0 (50.0; 52.0)	52.0 (51.0; 54.4)	69.5 (56.0; 76.25)	66.5 (61.8; 70.25)	54.0 (50.0; 66.5)	54.0 (51.0; 61.0)

Abbreviations: WMFT, Wolf Motor Function Test; FA, Functional Ability; URJC, Universidad Rey Juan Carlos; InTeO, Investigación en Terapia Ocupacional; UL, upper limb.

## Data Availability

Informed consent was obtained from all subjects involved in the study. Access to the data is subject to ethical and legal restrictions established by the Ethics Committees of General University of Alicante, Miguel Hernández University, and Rey Juan Carlos University. As stated in the informed consent form provided to participants, we ensured the confidentiality of all personal information collected through questionnaires and related sources. Data access requests can be directed to the corresponding author.

## Abbreviations

The following abbreviations are used in this manuscript:

WMFT	Wolf Motor Function Test
ABI	Acquired Brain Injury
TBI	Traumatic Brain Injury
UL	Upper Limb
FA	Functional Ability
CAULIN	European evidence-based recommendations for clinical assessment of upper limb in neurorehabilitation
ICF	International Classification of Function
URJC	Rey Juan Carlos University
InTeO	Investigación en Terapia Ocupacional (Research in Occupational Therapy group)
